# Tunable Cellular Localization and Extensive Cytoskeleton-Interplay of Reflectins

**DOI:** 10.3389/fcell.2022.862011

**Published:** 2022-06-23

**Authors:** Junyi Song, Chuanyang Liu, Baoshan Li, Liangcheng Liu, Ling Zeng, Zonghuang Ye, Ting Mao, Wenjian Wu, Biru Hu

**Affiliations:** ^1^ College of Liberal Arts Science, National University of Defense Technology, Changsha, China; ^2^ Logistics Center, National University of Defense Technology, Changsha, China

**Keywords:** reflectins, truncated peptides, intracellular localization, molecular evolution, cytoskeleton interaction

## Abstract

Reflectin proteins are natural copolymers consisting of repeated canonical domains. They are located in a biophotonic system called Bragg lamellae and manipulate the dynamic structural coloration of iridocytes. Their biological functions are intriguing, but the underlying mechanism is not fully understood. Reflectin A1, A2, B1, and C were found to present distinguished cyto-/nucleoplasmic localization preferences in the work. Comparable intracellular localization was reproduced by truncated reflectin variants, suggesting a conceivable evolutionary order among reflectin proteins. The size-dependent access of reflectin variants into the nucleus demonstrated a potential model of how reflectins get into Bragg lamellae. Moreover, RfA1 was found to extensively interact with the cytoskeleton, including its binding to actin and enrichment at the microtubule organizing center. This implied that the cytoskeleton system plays a fundamental role during the organization and transportation of reflectin proteins. The findings presented here provide evidence to get an in-depth insight into the evolutionary processes and working mechanisms of reflectins, as well as novel molecular tools to achieve tunable intracellular transportation.

## Introduction

Cephalopods (squid, octopus, and cuttlefish) have evolved a remarkable ability to manipulate light and alter their appearance *via* pigmentary and structural elements. In the uppermost layers of the dermis, chromatophore organs proportionally regulate coloration by stretching pigmentary sacs (Hanlon, 2007; Hanlon et al., 2009) while the structural coloration is produced by two classes of cells called iridocytes and leucophores, distributed on the periphery of iridocytes, Bragg reflectors are periodically stacked lamellae that present iridescence by multilayer interference (Tao et al., 2010; DeMartini et al., 2013a). Leucophores containing granular vesicles are responsible for producing bright white color by unselectively reflecting all incident light (Williams et al., 2019).

Interestingly, both periodically stacked lamellae in iridocytes (Tao et al., 2010; DeMartini et al., 2013a) and intracellular vesicles in leucophores (DeMartini et al., 2013b; Mäthger et al., 2013; Hanlon et al., 2018) are unique and composed of reflectin proteins. Reflectin proteins are exclusively expressed in cephalopods, and their members possess different numbers of canonical reflectin motifs in different sequential locations (Tao et al., 2010; DeMartini et al., 2015; Levenson et al., 2019). Guan et al. (2017) reported that reflectin motifs may be traced to a 24-bp transposon-like DNA fragment from the symbiotic bioluminescent bacterium *Vibrio fischeri*. Afterward, millions of years of self-replication and translocation of transposons forms a prosperous reflectin family. For example, in *Doryteuthis opalescens* (one representative of the most recently evolved Loliginid species), distinct reflectins, reflectins A1, A2, B, and C, have been identified and well-characterized (Tao et al., 2010; DeMartini et al., 2015; Levenson et al., 2019).

Numerous *in vitro* assays suggest that the dynamic condensation, folding, and hierarchical assembly of reflectin proteins play fundamental roles during the formation and regulation of those structural coloration elements in squids (DeMartini et al., 2015; Levenson et al., 2016; Levenson et al., 2019). Recently, Morse and his team have preliminarily defined reflectin proteins as a new group of intrinsically disordered proteins (IDPs) and suggested that transient liquid-liquid phase separation (LLPS) may dominate reflectin assembly (Levenson et al., 2019). The dynamic reflectin assembly properties and the intricate reflectin-based biophotonic systems have already inspired the development of various next-generation tunable photonic (Qin et al., 2013; Phan et al., 2015; Dennis et al., 2017) and electronic platforms and devices (Ordinario et al., 2014; Yu et al., 2014; Phan et al., 2016).

Despite the impressive breakthroughs achieved by the abovementioned references, knowledge about how reflectin molecules are spatially organized to form proteinaceous layers or droplets is still limited. Moreover, the limited existing information is mostly obtained from *in-tube* assays. It is necessary and interesting to explore reflectin properties in cells.

Recently, two different groups made groundbreaking attempts and used reflectins to modify mammalian cell function. The formation of protein-based photonic architecture endows engineered human cells with tunable transparency-changing and light-scattering capabilities by expressing reflectins in the human embryonic kidney (HEK-293) cells (Chatterjee et al., 2020; Ogawa et al., 2020). This is a milestone in the discovery of novel molecular tools to modify mammalian cells with new features (Tang et al., 2020).

However, a comprehensive understanding of reflectin intracellular behaviors is a prerequisite before exploring their application potential in the synthetic biology field one step further. A comparative analysis is not available to investigate the properties of these reflectins exclusively expressed by cephalopods due to the lack of homogenesis with other well-known proteins.

To overcome this drawback, we engineered HEK-293T cells to express either intact RfA1, RfA2, RfB1, or RfC. These proteins are effective controls to each other, with different self-assembly characteristics (Levenson et al., 2016; Levenson et al., 2019) and membrane-bound properties (Song et al., 2020). After transfection, reflectins are found to phase out from the crowded intracellular milieu, similar to what Atrouli (Chatterjee et al., 2020) and Junko (Ogawa et al., 2020) described. Beyond that, more dramatic is the exclusive distribution of RfA1 particles in the cytoplasm, while spherical RfC droplets only exist in the nucleoplasm. As an intermediate state, RfA2 and RfB1 condensates distributes in both the cytoplasm and nucleoplasm. To the best of our knowledge, this is the first report of discrepant intracellular features of reflectins.

As a result of molecular evolution, the number and localization of canonical motifs distributed throughout reflectins may dominate their functional heterogeneities. Though it is impossible to witness this evolution process that takes a million years, the work took advantage of reflectin programmable sequences and designed a series of RfA1 truncations to validate this hypothesis. By cutting off reflectin motifs (RMs) one by one from RfA1, the constitution and properties of RfA1 variants are more and more similar to the shorter natural reflectins, indicating a possible ancestor-descendant sequence and a tight relationship between motifs repetition and reflectin function.

Further, CoIP-MS was conducted to better understand the molecular mechanism underlying the localization preferences among RfA1 and variants. Molecular partners such as RAN and Nups were identified to facilitate the entrance of RfA1 truncations into nuclei. Except for mass spectrum data, both confocal observation and RNA-Seq analysis confirmed the extensive interplay between RfA1 and the cytoskeleton. Especially, RfA1 was observed to be enriched in the microtubule organizing center (MTOC), significantly affecting spindle organization and cell division at the transcriptomic level. This spatial enrichment can be abolished by nocodazole treatment, as it suppresses microtubule polymerization. Therefore, it is speculated that the enrichment of RfA1 may be the result of its orientated movement towards the microtubule minus-ends. This conjectural MT minus-end-directed movement is likely to recruit RfA1 beneath the cytomembrane in polarized epithelial cells, which should be essential for Bragg lamellae formation in iridocytes.

Briefly, the work presents many unprecedented experimental results to better understand the relations and distinctions among reflectins, the penitential mechanism underlying reflectin orientated intracellular transportation, and the formation of biophotonic structures. Moreover, their intracellular localization properties provide alternative options for manipulating the cytoplasmic/nucleic distribution of proteins of interest.

## Results

### Selective Intracellular Localization of Reflectin Proteins

**FIGURE 1:**
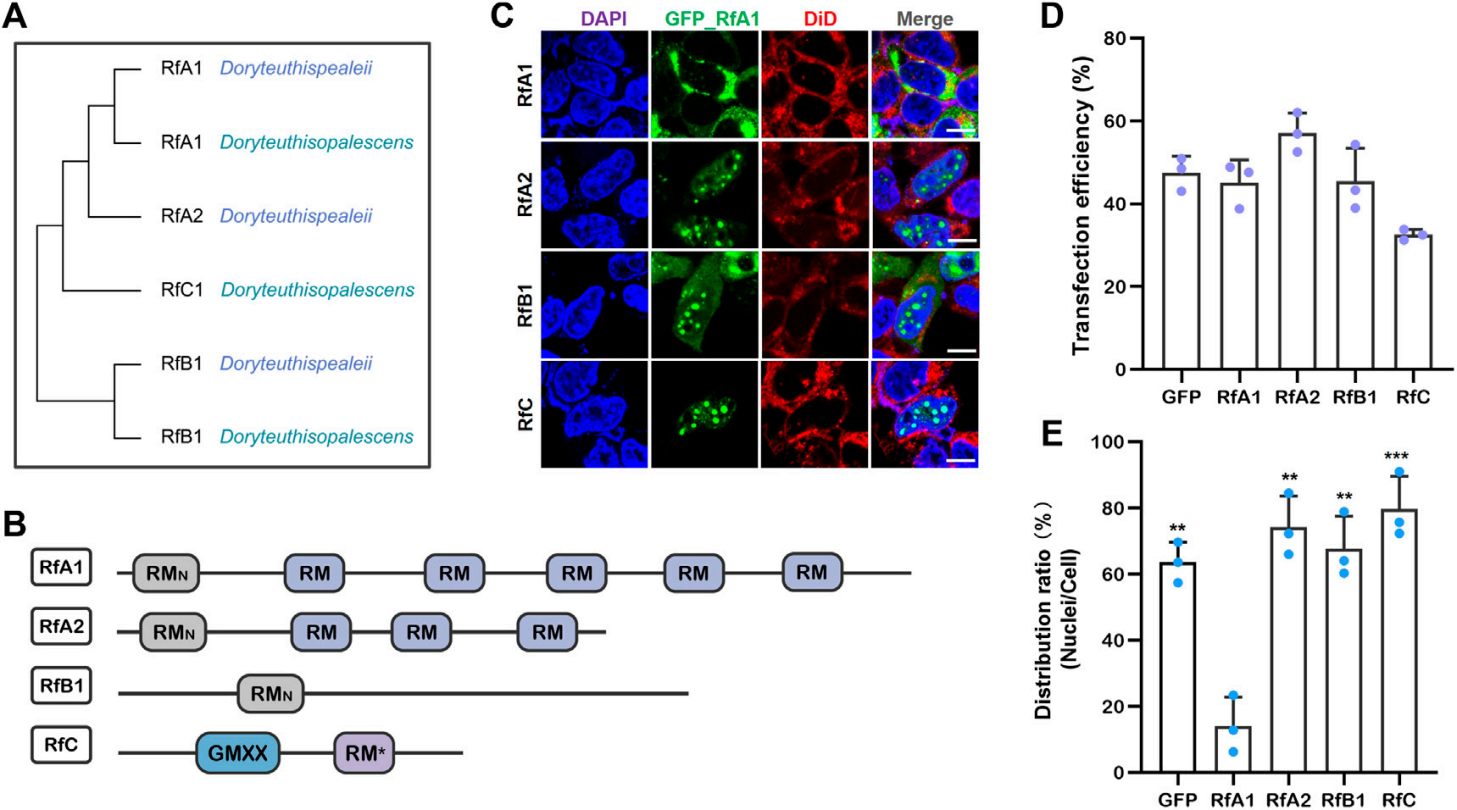
Transfection of reflectins induces protein condensate formation in selective intracellular regions. **(A)** Cladogram of RfA1, RfA2, RfB1, and RfC. **(B)** Schematic reflectins sequences. Reflectin motifs are designated by boxes, while reflectin linkers (RLs) are lines. **(C)** Confocal images of GFP_Reflectin condensates in the HEK-293T cells. Nuclei and membrane are stained with DAPI (blue) and DiD (red) respectively, scale bars = 10 μM. **(D)** Cell number statistics and quantification of transfection efficiencies of four reflectins. **(E)** Distribution ratio statistics of fluorescent intensity in transfected cells and their nuclei. Data are presented as mean values ± SD, for *n* = 3 independent experiments. *p*-values (from a two-sided unpaired t test):∗, *p* < 0.05; ∗∗, *p* < 0.01; ∗∗∗, *p* < 0.001; all groups are compared to RfA1.

RfA1, A2, and B1 possess different numbers and localization of two types of canonical reflectin motifs (see Figure 1A), the regular reflectin motifs {RMs [M/FD(X)5MD(X)5MDX3/4]}, and a N-terminal reflectin motif {RM_N_ [MEPMSRM-(T/S)MDF(H/Q)GR (Y/L) (I/M)DS (M/Q) (G/D)R (I/M)VDP (R/G)]} (see Figure 1B). Specifically, RfC is the shortest reflectin, containing a GMXX motif and RM*. The GMXX motif is a unique region of increased overall hydrophobicity composed of a four-amino acid repeat, and “X” represents less conserved locations within the repeat. Asterisk marked RM* of RfC contains substantial deviations in sequence not observed in any other reflectin motifs (Crookes et al., 2004) (see Figure 1B). The disorder tendencies of reflectins were calculated by PONDR (Xue et al., 2010), DISOclust (Ward et al., 2004; McGuffin, 2008), and ESpritz (Walsh et al., 2012). The possibility of forming protein droplets *via* liquid-liquid phase separation is also predicted by FuzDrop (Hardenberg et al., 2020). These calculations indicate a high disorder (see Supplementary Figure S1) and LLPS tendency (see Supplementary Figure S2) of four reflectin proteins. Reflectin proteins are introduced into the HEK-293T cells to verify these computational calculations.

After transfection and culturing for 12 h or longer, all RfA1, RfA2, RfB1, and RfC are phased out from the cytoplasm or nucleoplasm, which is consistent with computational predictions. However, their difference is more significant. For RfA1, protein condensates are exclusively enriched in the cytoplasm. In a sharp contrast, the perfect spherical RfC droplets are only located in the nucleoplasm. While the distribution of RfA2 and RfB1 seems to stay in a middle state, spherical protein droplets mainly exist in the nucleus, with the minority staying in the cytoplasm (see Figures 1C,E).

According to the report by Guan et al., reflectin genes in cephalopods come from a transposon in symbiotic *Vibrio fischeri* (Guan et al., 2017). The repetitions of conserved motifs in reflectin sequences are the results of transposon self-replication and translocation. Thus, it is speculated that diverse reflectin proteins with different motifs stay at different evolution stages or represent different evolution directions.

From this point, RfC with only a GMXX motif and RM* may be the earliest appeared ancestor, while RfA1 with a RM_N_ and five RMs is the further evolved offspring protein. Based on this consideration, truncated variants were constructed in the work by cutting off the RMs from the RfA1 one by one, to make their composition comparable to shorter natural reflectins. The dynamic intracellular performance of these RfA1 variants were then investigated.

### Intracellular Localization Preferences of RfA1 Variations

**FIGURE 2:**
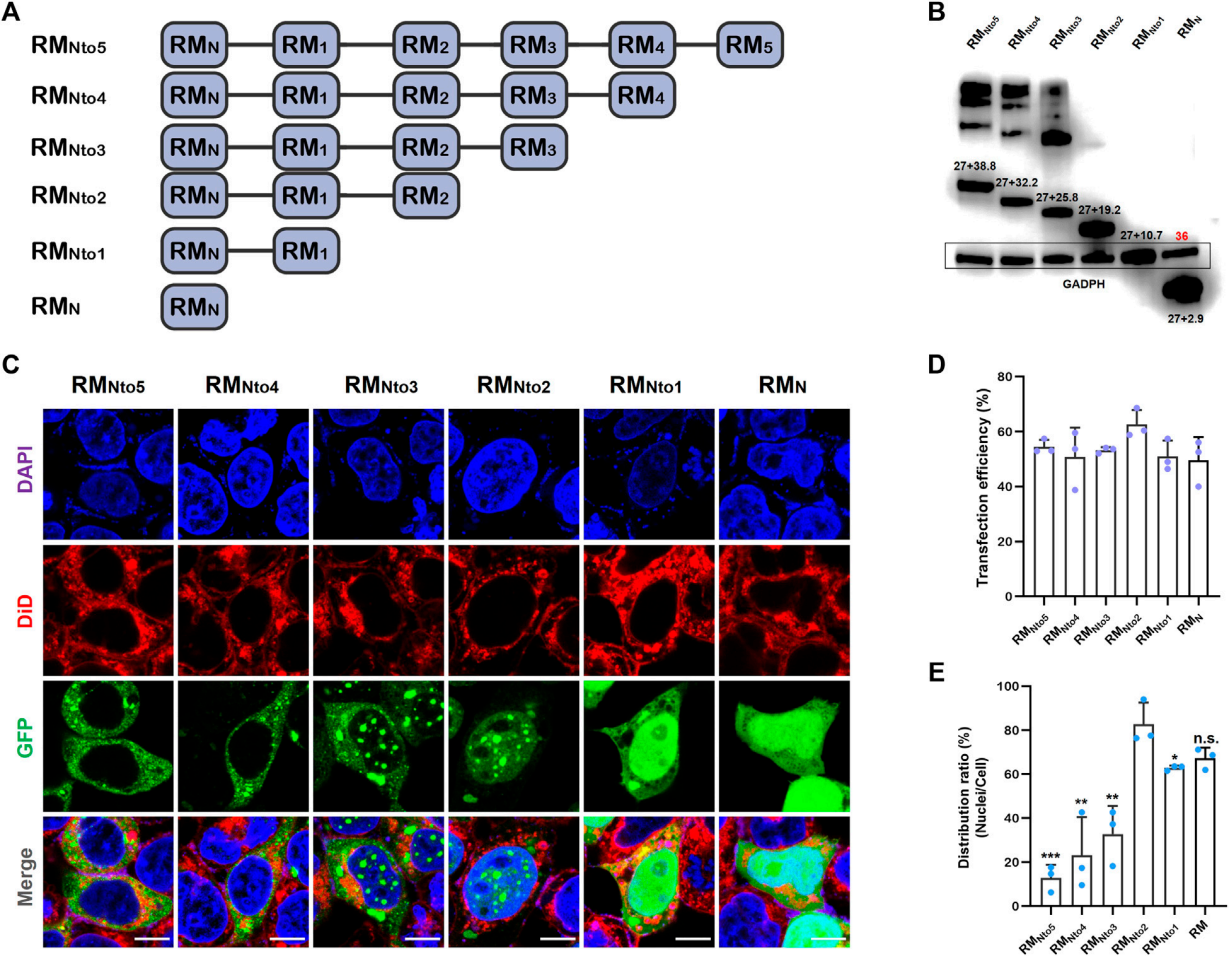
Expression of RfA1 variants in the HEK293T cells. **(A)** Schematics of RfA1 truncated variants. **(B)** Expression of RfA1 variants validated by Western blotting and indicated by anti-GFP antibodies. The molecular weight of EGFP is 27 kDa and GADPH is 36 kDa. **(C)** Cyto-/nucleo-localization preferences of truncated RfA1 derivatives. Nuclei and membrane are stained with DAPI (blue) and DiD (red), RfA1-derived peptides are indicated by tandem EGFP (green), scale bars = 10 μM. **(D)** Cell number statistics and quantification of transfection efficiencies of four reflectins. **(E)** Distribution ratio statistics of fluorescent intensity in transfected cells and their nuclei. Data are presented as mean values ± SD, for *n* = 3 independent experiments. *p*-values (from a two-sided unpaired t test): ∗, *p* < 0.05; ∗∗, *p* < 0.01; ∗∗∗, *p* < 0.001; All groups are compared to RM_Nto2_.

Six pairs of primers were designed to clone RfA1 truncations (see Supplementary Table S1). The PCR products responsible for coding six peptides, including RM_Nto5_, RM_Nto4_, RM_Nto3_, RM_Nto2_, RM_Nto1_, and RM_N_ (schematics shown in Figure 2A), were subsequently ligated to vector pEGFP-C1. After transfection, the expressions of GFP-tagged RfA1 truncations were validated by an anti-GFP Western blotting (Figure 2B).

Cells were harvested and RfA1-binding protein was extracted by an immunoprecipitation kit (Sangon) 24 h after transfection. GFP monoclonal antibody was used to validate the expression of GFP-tandem RfA1 truncations, while GADPH monoclonal antibody was employed to adjust the sample-loading quantity. In Figure 2B, the intensities of 36kD GADPH are almost the same among different lanes, implying that the sample loadings are normalized. The numbers labeled above bands are molecular weights calculated by ExPASy ProtParam software, consistent with the sums of GFP and RM_NtoX_ (*X* = 0, 1, 2, 3, 4, and 5). Moreover, for longer variants RM_Nto5_, RM_Nto4_, and RM_Nto3_, there are significant smears with very large molecular weights. Meanwhile, the lanes of shorter variants are quite clean. It indicates that RfA1 truncations with more motifs tend to tightly interact with other cellular proteins, which is discussed in detail in the next section.

On the other hand, a confocal image system is employed to investigate the intracellular localization of GFP-tagged RfA1 truncations (see Figure 2C). The nuclei and membrane are stained with DAPI (blue) and DiD (red), and RfA1-derived peptides are indicated by tandem EGFP (green). For cells transfected with pEGFP-C1-RM_N_ and pEGFP-C1-RM_Nto1_, though the peptide condensation seems absent, a remarkable enrichment of the green fluorescence signal in the nuclei areas appears. Amazingly, this nucleus-enrichment is executed more thoroughly by RM_Nto2_, along with the recurrence of proteinaceous droplets. With increase in conserved motifs, RM_Nto3_ are found to be distributed in both cyto- and nucleo-plasm. For longer derivatives with more motifs, condensates of RM_Nto4_ and RM_Nto5_ are blocked out from nuclei and located exclusively in cytoplasmic localization, extremely resembling their template RfA1.

Briefly, RM_Nto2_ is most similar to RfC among all variants, which forms condensates exclusively in the nucleoplasm. More significantly, RM_Nto2_ is also a definite boundary: truncations shorter than RM_Nto2_ tend to enrich in the nuclei but cannot form droplets; derivatives longer than RM_Nto2_ escape from the nuclei and accumulate and condensate into droplets in the cytoplasm.

Note smears in PAGE lanes of RM_Nto3_, RM_Nto4_, and RM_Nto5_ in Figure 2B. Those are not sample contaminations or non-specific labeling. Subsequent CoIP-MS results reveal that various cellular components tightly interact with RfA1 variants, which assists their phase separation and diverse cellular localization.

### Identification of RfA1/RM_Nto2_ Binding Proteins

A monoclonal antibody of GFP was prepared and a CoIP-MS approach was used to identify protein-protein interactions, thus exploring the mechanism underlying phase separation and distinguished distribution of RfA1 and RM_Nto2_.

**FIGURE 3:**
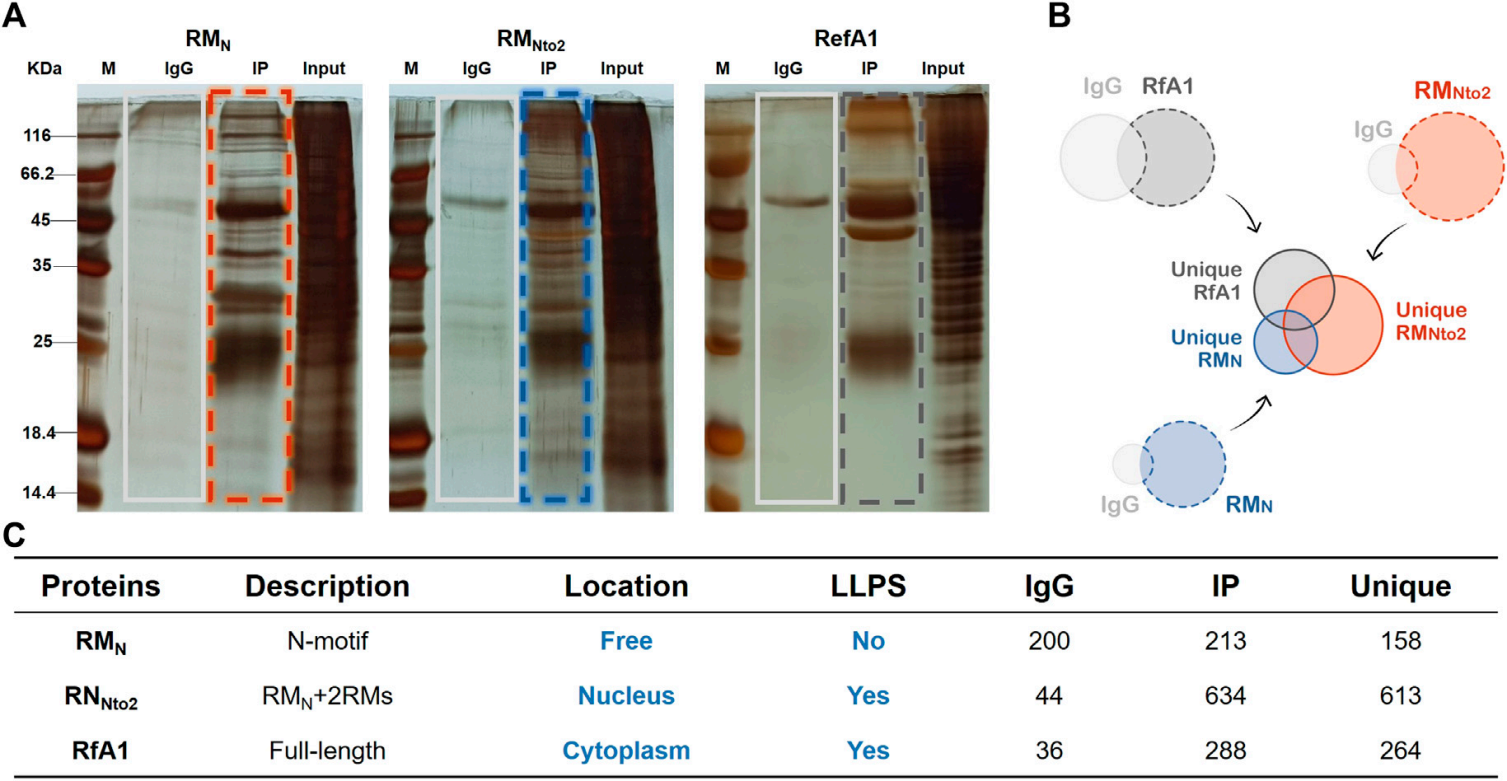
Identification of interaction proteins targeted to RfA1 and RM_Nto2_. **(A)** Silver staining of IP products, whole IP bands (marked by boxes) are sent to LC-MS. **(B,C)** PPI summary. Unique (RM_N_) = IP(RM_N_)-IP(RM_N_)∩IgG (RM_N_); Unique (RM_Nto2_) = IP(RM_Nto2_)-IP(RM_Nto2_)∩IgG (RM_Nto2_); Unique (RfA1) = IP(RfA1)-IP(RfA1)∩IgG (RfA1).

Aforementioned, there must be a massive interaction between RfA1 variants and host cell proteins, resulting in smears in Figure 2B. These smears are not meaningless miscellaneous proteins, but hybrid aggregates recruited by RfA1 derivatives. Therefore, valuable information would be lost if only one single band was collected and analyzed. However, the collection of multiple bands as a mixed sample will inevitably bring in disturbing data. Based on these considerations, cells that express RM_N_ are selected as a control group instead of GFP-expressed cells since RM_N_ presents no significant phase separation or selective distribution behavior. As shown in Figure 3A, the IgG and IP bands of RM_N_, RM_Nto2_, and RfA1 are collected and sent for mass-spectrum analysis. After that, proteins specifically interacting with RM_N_, RM_Nto2_,and RfA1 are screened with a simple mathematical logic: Unique (RM_N_) = IP(RM_N_)-IP(RM_N_)∩IgG (RM_N_); Unique (RM_Nto2_) = IP(RM_Nto2_) -IP(RM_Nto2_)∩IgG (RM_Nto2_); Unique (RfA1) = IP(RfA1)-IP(RfA1)∩IgG (RfA1) (see Figures 3B,C).

**FIGURE 4:**
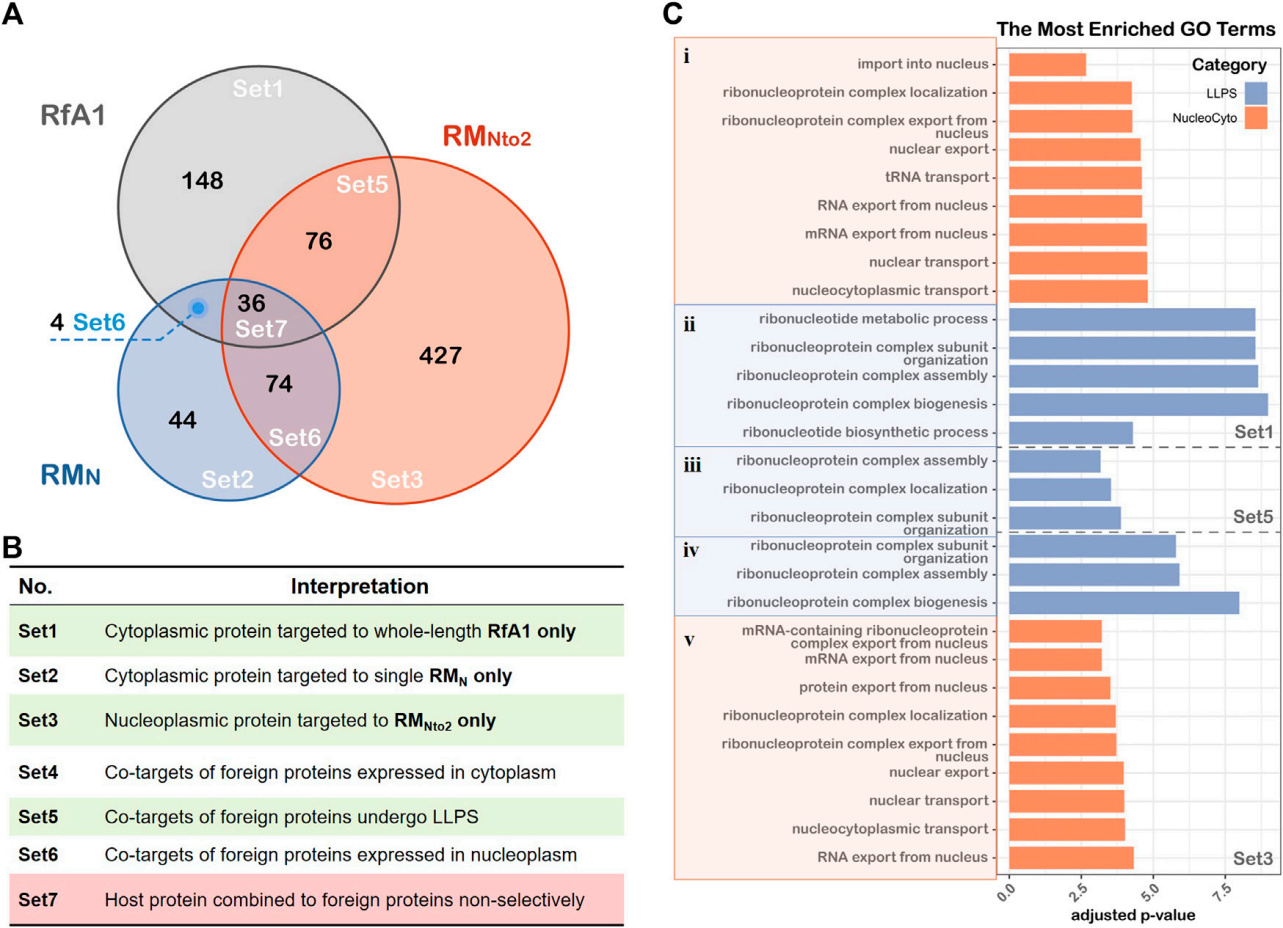
GO biological processes annotation of proteins associated with LLPS and cellular localization. **(A)** Venn diagram showing the results of LC-MS, the graph is divided into Set1∼Set7 based on inclusion-exclusion principle of datasets. **(B)** Interpretations of different sets in the Venn graph. **(C)** Gene lists in Set1, Set5 and Set3 are converted to ENTREZ ID, and R package clusterProfiler is used to perform Gene Ontology annotation. LLPS and cellular localization-associated BP terms are showed in the figure, and the thresholds set as log_10_ (adjusted *p*-value) < −2.5.

To further identify proteins which are responsible for intracellular localization or the formation of condensates, Unique (RM_N_), Unique (RM_Nto2_), and Unique (RfA1) are divided into subsets according to the venn diagram in Figure 4A. According to the venn diagram, overlaid sets Set4/6/7 are eliminated as backgrounds. Within Set1 are the proteins combined into whole-length RfA1 and contribute to the phase separation and cytoplasmic localization. Besides, proteins in Set3 promote the importation of RM_Nto2_ into nuclei and its phase out from the nucleoplasm. Co-targets of RM_Nto2_ and RfA1 are sorted into Set 5.

Gene Ontology (GO) annotation is then performed to investigate biological processes associated with interacting proteins of RfA1 and RM_Nto2_. Figure 4C shows the items of interest with an adjusted *p*-value<0.05 (see Supplementary Table S2 for gene lists and Supplementary Figure S3 for PPI networks).

Taking Set1-i for example, since RfA1 binds to proteins like HNRNPA1 (Matthew Michael et al., 1995; Roy et al., 2014), SRSF1 (Hautbergue et al., 2017), and RAN (Connor et al., 2003; Güttler and Görlich, 2011) and participates in processes including “protein export from nucleus”, RfA1 condensates are exclusively distributed in the cytoplasm. Oppositely, RANBP2 (Zhang et al., 2010) and NUPs (Cautain et al., 2015) in Set3-v facilitate the importation of RN_Nto2_ into nuclei.

One remarkable common point among Set1-ii, Set5-iii, and Set3-iv is the involvement of “ribonucleoprotein complex” or “ribonucleotide metabolic”. The interaction with ribonucleoprotein complexes brings in a massive interplay with proteins and mRNA (Sfakianos et al., 2016; Mukherjee et al., 2019), leading to almost inevitable phase separation. This is consistent with the results in Figure 2C. However, the involvement of the ribonucleoprotein complex makes the situation more elusive. It is equivocal whether RfA1/RN_Nto2_ molecules gather together and are phased out from the surroundings on their own, or they are drawn into the formation processes of ribonucleoprotein complex bodies.

Moreover, PPI information also demonstrates that RfA1 extensively interacts with actin and actin-binding proteins (ABPs) (Supplementary Table S3 for gene lists and Supplementary Figure S4 for PPI networks). These proteins are in charge of various biological processes, including actin polymerization, nucleate assembly of new filaments, and promotion of elongation (Uribe and Jay, 2009; Pollard, 2016). Considering the critical role of actin filaments in forming protrusive structures such as lamellipodia and ruffles (Mejillano et al., 2004; Chhabra and Higgs, 2007; Weiner et al., 2007; Innocenti, 2018), the results presented here highly suggest RfA1’s potential contribution in the establishment and regulation of Bragg lamellae *via* its collaboration with the actin system. RfA1 can anchor at the inside of the Bragg lamella by cooperating with actin and ABPs, or even participate in the organization of the actin network, which regulates cell morphogenesis.

### Verification of Co-localization of RfA1 and Actin System

Actin should be indispensable for either the establishment or regulation of delicate Bragg reflectors in iridocytes. However, so far, except for the Co-IP results presented in this work, no evidence has been issued about the interaction between reflectin proteins and the cytoskeleton. As a preliminary start, we stained the HEK-293T cells with ActinRed 555 (Thermo) to trace the actin networks and verified the potential relationship between RfA1 and the cytoskeleton.

**FIGURE 5:**
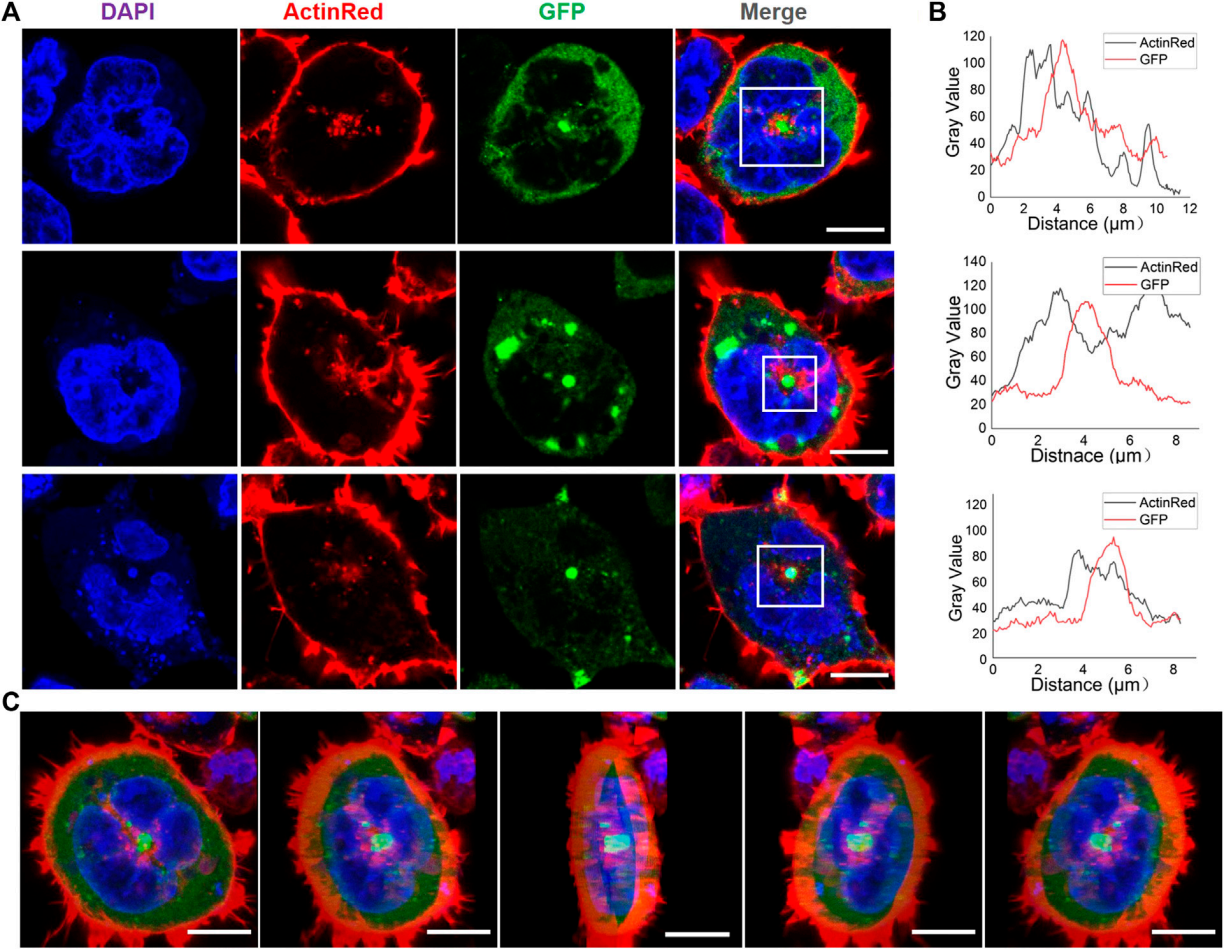
Appearance of spherical RfA1 condensates in the center or on the central axis of transfected cells. **(A)** RfA1 condensates located in the center or on the central axis of cells. Nuclei and actin are stained with DAPI (blue) and ActinRed 555 (red), RfA1 are indicated by tandem EGFP (green), scale bars = 10 μM. **(B)** Statistics of fluorescence signal distribution in selected areas. **(C)** 3D images of a transfected cell, with green RfA1 condensates located in the center, scale bars = 10 μM (see Supplementary Movie S2).

One notable trouble is the ubiquity of the actin cytoskeleton. It is challenging to judge the co-localization of RfA1 and actin (see Supplement Movie. 1mmc1). However, after viewing thousands of cells, an interesting scenario emerges. For plenty of transfected cells, there is a bright green sphere in the center or on the central axis, which are condensates of RfA1 (see Figure 5A for GFP channel, Supplementary Figure S5). These central localized protein condensates are not found in RfA2, RfB1, or RfC transfected cells. Around these spherical condensates, the red signal of stained actin is also significantly stronger than in other areas (see Figure 5A for ActinRed channel). The fluorescence statistics of selected areas in Figure 5B show an almost synchronized fluctuation of ActinRed and GFP signals, indicating their co-localization (see Supplementary Table S4 for raw data).

### RfA1 Interacts With Microtubules and is Highly Enriched in the Microtubule Organization Center

**FIGURE 6:**
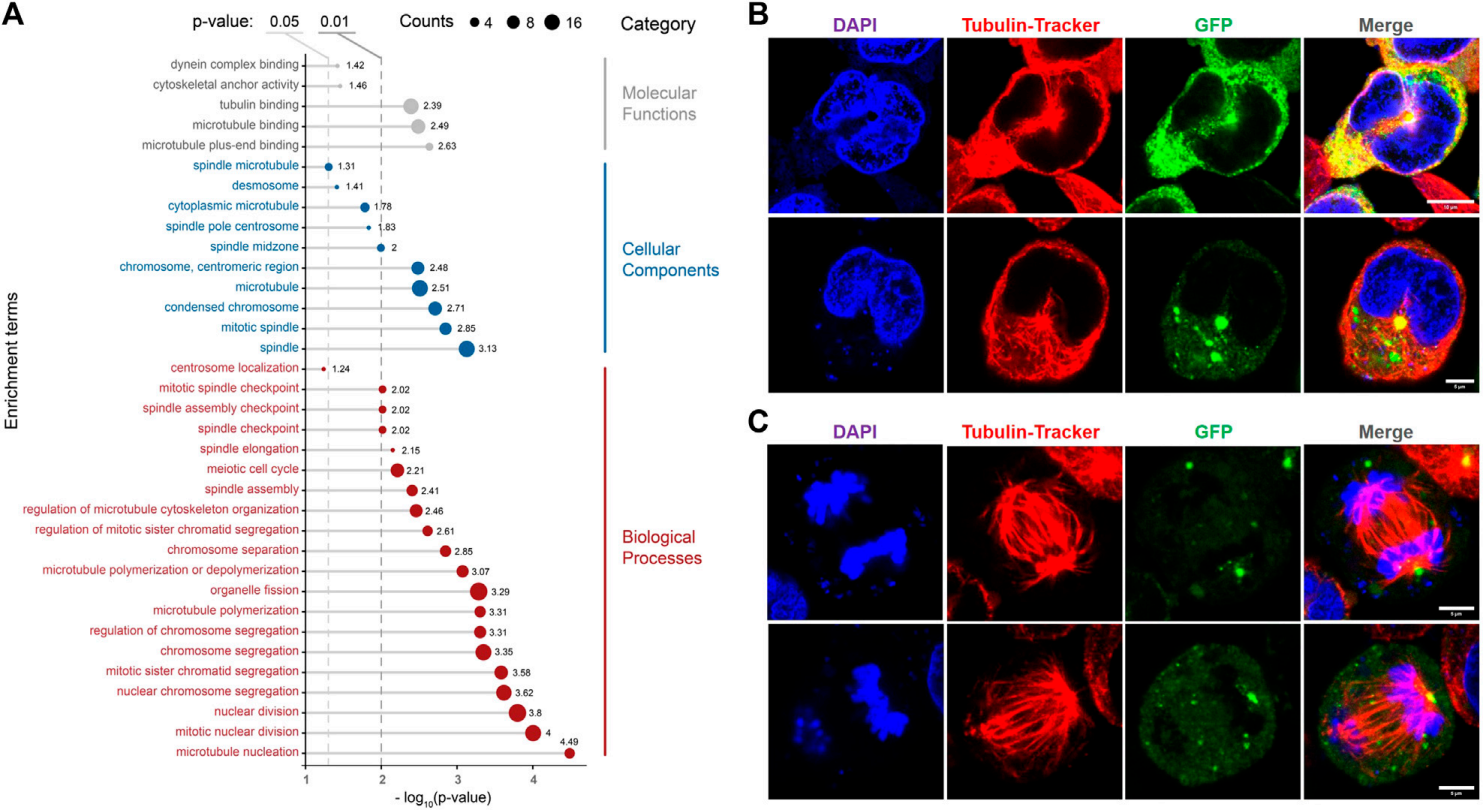
Effects of RfA1 on microtubule and mitosis. **(A)** GO annotation of DEGs between pEGFP-C1 transfected cells and pEGFP-C1-RfA1 transfected cells. Genes are converted to ENTREZ ID and R package clusterProfiler is used to perform GO annotation. Biological Process, Cellular Component and Molecular Function terms are shown in the figure, and the threshold was set as the *p*-value< 0.05. **(B)** RfA1 co-localization with microtubule networks in the center, in interphase cells. Nuclei and microtubule are stained with DAPI (blue) and Tubulin-Tracker Red (Beyotime), RfA1 are indicated by tandem EGFP (green), scale bars = 10 μM. **(C)** RfA1 located at the spindle center, in late mitosis cells. Nuclei and microtubule are stained with DAPI (blue) and Tubulin-Tracker Red (Beyotime), RfA1 is indicated by tandem EGFP (green), scale bars = 5 μM (see Supplementary Movies S3, S4).

Except for the fluorescent observation of the actin system, RNA-seq results also strongly suggested the tight relationship between RfA1 and the cytoskeleton. Cells transfected with pEGFP-C1-RfA1 and no-load pEGFP-C1 (set as control) are harvested for sequencing. After Metascape screening (Zhou et al., 2019), 461 differentially expressed unigenes are identified in RfA1 expressed cells (Supplementary Table S5). GO analysis is then performed to annotate differentially expressed genes (DEGs). Figure 6A shows the selected items of interest. Genes evolved in biological processes such as “regulation of microtubule cytoskeleton organization,” “microtubule polymerization or depolymerization,” and “microtubule nucleation” respond to RfA1 expression. Correspondingly, these microtubule-relevant genes are also sorted into cellular component items, including “spindle microtubule,” “spindle pole centrosome,” “mitotic spindle,” and “spindle” (Wittmann et al., 2001; Foley and Kapoor, 2013), which indicates the tight relationship between RfA1 and microtubules.

The analysis based on RNA-Seq is further confirmed by confocal observation. After staining the fixed cells with Tubulin-Tracker Red (Beyotime), RfA1 condensates are significantly enriched at MTOC, located around the centrosome (see Figure 6B). For cells staying in late mitosis, RfA1 condensates are no longer located in the central areas but seem to chase the movements of daughter centrosomes (see Figure 6C and Supplementary Movies S3, S4 for Z-axis scanning). This significant enrichment of RfA1 at MOTC can be severely abolished by nocodazole treatment (data not shown). More directly, except for fluorescent colocalization observation, RfA1 is also detected to bind to tubulin subunits TBB2B, TBB4A, and TBB4B (Supplementary Table S6) by CoIP, suggesting physical interaction between RfA1 and microtubules.

## Discussion

**FIGURE 7:**
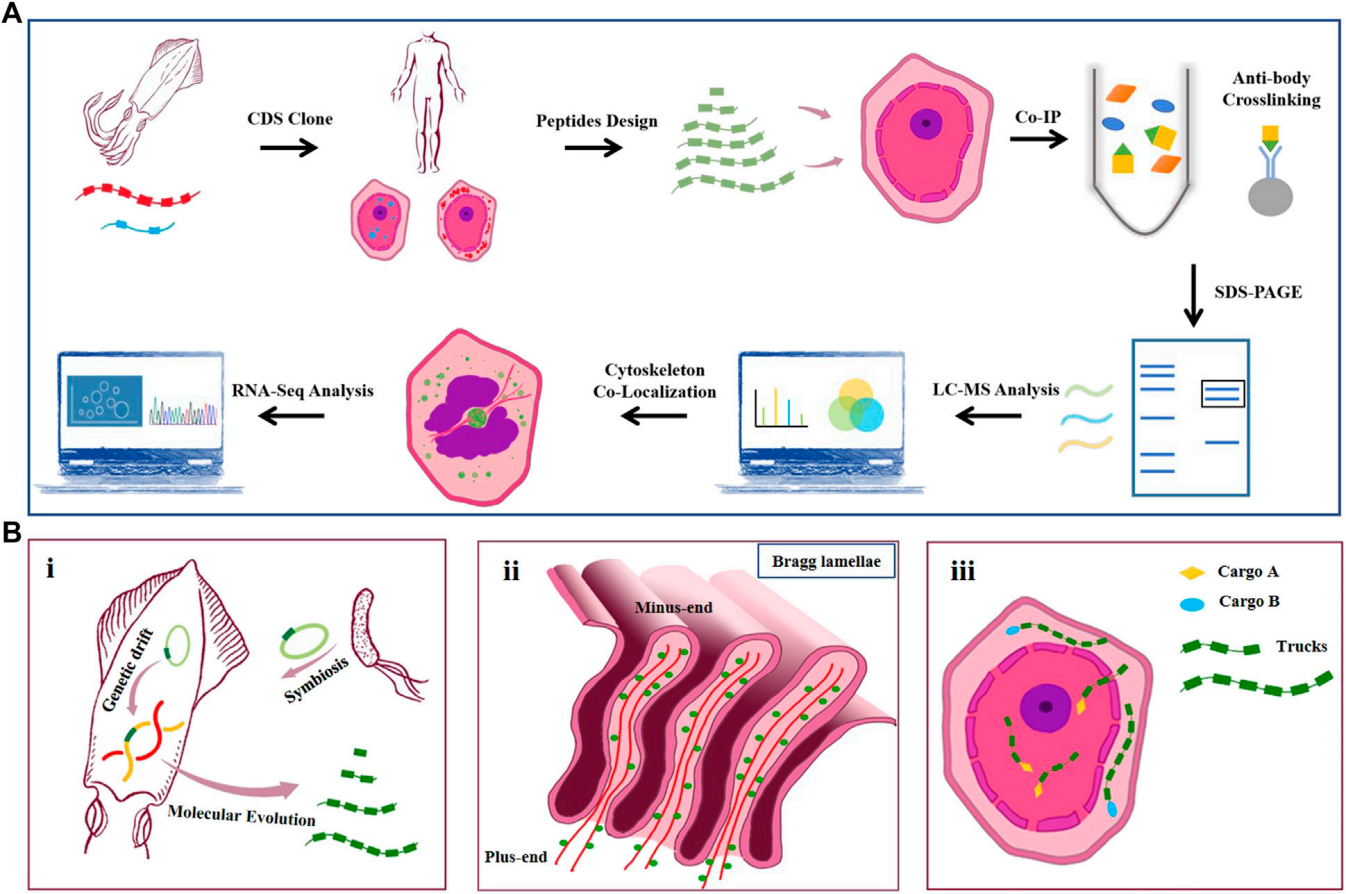
**(A)** Schematic of the workflow. CDS of squid reflectin proteins are obtained from NCBI. Codons are optimized according to mammal cell expression preference and introduced into HEK-293T cells by lipofection. RfA1 truncated variants are designed and cloned into pEGFP-C1 vectors. Anti-GFP monoclonal antibody is used to pull down GFP-tagged RfA1 variants and their binding proteins. Immunoprecipitates are separated using SDS-PAGE. Gel lanes are digested with trypsin for LC-MS/MS analysis. Colocalization of RfA1 and actin cytoskeleton is confirmed by confocal microscope. RNA-Seq is employed to globally investigate the influence of RfA1 expression on cells. **(B)**. Illustration for hypothetically models. i. Origin and evolution of reflectin proteins. ii. Microtubule minus-end-directed transportation of reflectin. iii. Schematic for RfA1 derivatives as programmable and oriented cargo carriers.

In summary, the work realized the expression of four natural reflectins (RfA1, RfA2, RfB1, and RfC) and six engineered RfA1 in cells and found their tunable intracellular localization. To the best of our knowledge, this is the first report of discrepant intracellular features of reflectins and their derivatives. Further, CoIP-MS was conducted to better understand the molecular mechanism underlying the localization preferences among RfA1 and variants. Moreover, both confocal observation and RNA-Seq analysis confirmed the extensive interplay between RfA1 and the cytoskeleton, providing evidence to delineate the role of RfA1 during Bragg lamellae establishment (Figure 7A). There are multiple reasons to support the significance of the work.

Firstly, the cyto-/nucleoplasmic localization preference presented here could be evidence for the molecular evolution of reflectin proteins. According to Guan’s report, reflectin genes in cephalopods come from a transposon in symbiotic *Vibrio fischeri* (Guan et al., 2017). Afterward, millions of years of replication and translocation of this transposon forms reflectins with different lengths, motifs, and properties (Tao et al., 2010; DeMartini et al., 2015; Levenson et al., 2017; Levenson et al., 2019). In the work, RfA1 consists of one RM_N_ and five RMs exclusively enriched in cytoplasm, while shorter RfA2, RfB1, and special RfC contains only one GMXX and an RM* tends to stay in nuclei. By truncating RfA1 gradually, shorter peptide derivatives (e.g., RM_Nto2_) localize in nuclei, behaving exactly similar to simpler reflectins. From this point of view, the repetition numbers of RMs (as basic units) accurately determine the disparate properties (e.g., intracellular localization) of RfA1, A2, B1, and C. Being a subordinate element and of evolutionary origin, the 24-bp transposon-like DNA fragment is expected to be the root source of reflectin diversification, which makes our finding a strong clue to support Guan’s evolution hypothesis. Besides, earlier and simpler reflectins with fewer motifs tend to stay in the nuclei since there are abundant molecular tools available in the nuclei to evaluate, to manipulate, or even to edit this passage of alien DNA sequence. As for more complex reflectins such as RfA1, they are excluded from the nuclei and participate in other functional processes in the cytoplasm, like establishing and regulating Bragg lamellae [see Figure 7b(i)].

Secondly, the nucleus entry of short reflectins and centrosome-enrichment of RfA1 delineate their potential organization mode in iridocytes, which lay the material foundation for Bragg lamellae establishment. 1) Seeing from the longitudinal section, the pore at the bottom of Bragg lamellae is approximately equal to the size of the ciliary pore and nuclear pore (see Figure 7b(ii)). Though knowledge about protein transportation between the cytoplasm and Bragg lamellae is absent, the entrance of RfA1 truncations and short reflectins into nucleoplasm is observed. Here, RAN and Nups are identified to mediate the access of RfA1 variants into the nuclei. Presumably, the entry of reflectins into Bragg lamellae follows a similar mechanism, under the guidance of similar components (RAN, Nups, etc.). 2) Both CoIP-MS analysis and the RNA-Seq survey reveal the extensive interactions between RfA1 and the cytoskeleton. Actin and ABPs were identified to be combined with RfA1. Considering the critical role of actin filaments in the formation of protrusive structures such as lamellipodia and ruffles (Mejillano et al., 2004; Chhabra and Higgs, 2007; Weiner et al., 2007; Innocenti, 2018), the results presented here highly suggest RfA1’s potential contribution to the establishment and regulation of Bragg lamellae *via* its collaboration with the actin system. Moreover, RfA1 is observed to enrich at MTOC and co-localize with centrosomes. This spatial enrichment of RfA1 can be effectively abolished by nocodazole treatment. Since nocodazole suppresses the microtubule polymerization, this phenomenon suggests that the integrity of microtubule may be fundamental to RfA1 orientated transportation. Therefore, it is speculated that the orientated trafficking along the MT networks should be the critical property for RfA1 to work in Bragg lamellae. For the HEK-293T cells, microtubule minus-ends are located at or around the centrosome, guiding the movement of RfA1 to MTOC. However, microtubules are oriented apico-basally in polarized epithelial cells, with their minus-ends directed towards the apical side of the cells (Sugioka and Sawa, 2012; Glotfelty et al., 2014). This is partly verified when we perform new studies on the MDCK cells, which oppose apico-basal paralleled microtubule networks (data not shown). To the best of our knowledge, this is the first experiment to depict the potential mechanism underlying Bragg lamellae formation [see Figure 7B(ii)]. Although there is a huge difference between the mammalian cells and squid cells, the evolutionary conservatism of actins among algae, amoeba, fungi, and animals (Dominguez and Holmes, 2011; Gunning et al., 2015) eliminates this drawback.

Thirdly, reflectins are natural block copolymers and programmable biomaterials. Their intracellular localization properties provide alternative options for the manipulation of the cytoplasmic/nucleic distribution of proteins of interest. If we take GFP as a molecular cargo, reflectin derivatives can be regarded as intelligent vehicles to transport cargoes to pre-selected destinations [see Figure 7B(iii)]. Compared to the commonly-used NLS sequence, our programmable tools also have better scalability and modularity. The distribution of selected proteins can be easily altered by linking programmable tools to proteins (merely changing the numbers of parts), making them present only in the cytoplasm or nuclei. Using NLS, however, leads to a one-way trip into the nucleus. As a quick example, our tools might hypothetically achieve a switch of “cytoplasmic/nucleic distribution” at the protein-protein interaction level by linking different lengths of our parts to drug-/light-induced protein dimerization pairs. Further studies have already started in our research group (unpublished yet).

## Methods

### Construction of Recombinant pEGFP-C1 Vectors

The nucleotide sequence of *D. (Loligo) pealeii* reflectin A1 (RfA1) (Genbank: ACZ57764.1) and *D. (Loligo) Opalescens* reflectin C (Genbank: AIN36559.1) were optimized for human cell expression, and then synthesized and sequencing-identified by Sangon Biotech^®^ (Shanghai, China). Primers (F-GAATTCTAT GAA​TAG​ATA​TTT​GAA​TAG​ACA; R-GGATCCATACATATGATAATCATAATA ATTT) were designed to introduce *EcoR I* and *BamH I* cutting sites, so the modified RfA1 CDS can be constructed into pEGFP-C1 *via* a standard restriction-enzyme cloning process. As for the truncated RfA1 derivatives, six pairs of primers were coupled. For example, if RM_N_-F and RM_3_-R primers were selected, then a nucleotide sequence responsible for the coding of RM_N_-RL_1_-RM_1_-RL_2_-RM_2_-RL_3_-RM_3_ (simplified as RM_Nto3_ in the work) would be obtained after PCR. Meanwhile, 5′ GCA​TGG​ACG​AGC​TGT​ACA​AG 3′ and 5′ TTATGATCAG- TTATCTAGAT 3’ were added to F-primers and R-primers during the synthesis of the primer, respectively, which enables the sequences to be ligated to pEGFP-C1 by Ready-to-Use Seamless Cloning Kit from Sangon Biotech^®^ (Shanghai, China).

### Growth and Transfection of Human Cells

The HEK-293T cells (ATCC^®^, CRL-3216^TM^) were cultured on plastic dishes in Dulbecco’s Modified Eagle Medium (DMEM, Gibco^TM^) supplemented with 10% fetal bovine serum (FBS, Gibco^TM^) at 37°C under 5% CO_2_. One day before transfection, cells were seeded at ∼33% of the confluent density for the glass bottom dishes from Cellvis (California, United States) and grown for another 24 h. Then a transfection mixture containing Lipofectamine 3,000 (Thermo Scientific) and recombinant vectors were added to the medium and incubated for ∼16–∼24 h. For CCK-8 test, 1 × 10^4^ cells were seeded into each hole of 96-well plates 1 day before transfection, then transfected with recombinant vectors and incubated for another 24 h. After that, 10 μl CCK-8 solution was added to the wells for a chromogenic reaction of ∼2–∼4 h. OD_450_ was detected by Multiskan FC (Thermo Scientific).

### Fluorescence Microscopy of Stained Cells

Transfected HEK-293T cells grown in Cellvis plastic dishes were first fixed with 4% paraformaldehyde at room temperature for 30 min, then stained with DiD or ActinRed (diluted in 0.5% Triton X-100 PBS) for ∼30 min after PBS rinses. After washing off the fluorescent dye with PBS, fixed cells were embedded in DAPI-Fluoromount (Beyotime, Shanghai, China) and characterized with a Leica TCS SP8 imaging system in fluorescence imaging mode. The resulting images were analyzed with ImageJ (Java 1.8.0_172/1.52b) (Schindelin et al., 2012).

### Online Computational Prediction Tools

Disorder prediction of RfA1 and derivatives was conducted with PONDER^®^ (http://www.pondr.com/), DISOclust (http://www.reading.ac.uk/bioinf-/DISOclust/) and ESpritz (http://protein.bio.unipd.it/espritz/). The probability of liquid-liquid phase separation was calculated by FuzPred (http://protdyn-fuzpred.org/).

### SDS-PAGE, Western Blotting

Total proteins of cells were extracted by using the immunoprecipitation kit (Sangon). Protein samples were separated by SDS-PAGE and transferred to polyvinylidene fluoride (PVDF) filter membranes (Millipore, United States) for immune-hybridization. After 1 h of blocking in PBST (phosphate-buffered saline containing 0.05% Tween-20 and 2.5% BSA), the membranes were incubated with one of the following primary antibodies at corresponding concentration: anti-GFP mouse monoclonal antibody (Sangon), and anti-GAPDH mouse monoclonal antibody (Sangon). Subsequently, band visualization was performed by electro-chemiluminescence (ECL) and detected by the Digit imaging system (Thermo, Japan).

### In-Gel Digestion

For enzymolysis in protein gels (the gel strip, protein solution, and protein freeze-dried powder), a clean blade was used to dig out the strip of interest and cut it into 0.5–0.7 mm cubes. After decolorizing with test stain/silver stain decolorizing solution, the rubber block was washed with 500 μl acetonitrile solution three times until the rubber particles become completely white. Add 500 μl of 10 mM DTT to a water bath at 56°C for 30 min. Use low-speed centrifugation for a reduction reaction. Then add 500 μl decolorizing solution and mix at room temperature for 5–10 min Wash the gel spots once, and centrifuge at low speed to discard the supernatant After quickly adding 500 μl 55 mM IAM, place it in a dark room at room temperature for 30 min. Centrifuge at a low speed for an alkylation reaction. Then add 500 μl of decolorizing solution and mix at room temperature for 5–10 min After washing the gel spots once, centrifuge at low speed, and discard the supernatant; then add 500 μl of acetonitrile until the colloidal particles become completely white, and vacuum dry for 5 min. Add 0.01 μg/μl trypsin according to the gel volume. After an ice bath for 30 min, add an appropriate amount of 25 mM NH_4_HCO_3_ into pH8.0 enzymatic hydrolysis buffer, and enzymatically hydrolyze overnight at 37°C. After enzymolysis, add 300 μl extract with sonicate for 10 min. Centrifuge at a low speed, to collect the supernatant. After repeating the process twice, combine the obtained extracts and perform a vacuum dry.

### Zip-Tip Desalting

Dissolve the sample with 10–20 μl 0.2% TFA, and centrifuge at 10,000 rpm for 20 min. Then wet the Ziptip 15 times with a wetting solution and equilibrate with a balance solution 10 times. Finally, inhale the sample solution for 10 cycles and blow 8 times with the rinse liquid. Note: To avoid cross-contamination, aliquot 100 μl flushing solution per tube and use a separate tube for each sample. Add 50 μl eluent to a clean EP tube, and pipette it repeatedly to elute the peptide. Finally, drain the sample.

### LC-MS/MS Analysis

The peptide samples were diluted to 1 μg/μl on the machine buffet. Set the sample volume to 5 μl and collect the scan mode for 60 min. Scan the peptides with a mass-to-charge ratio of 350–1200 in the sample. The mass spectrometry data was collected using the Triple TOF 5600 + LC/MS system (AB SCIEX, United States). The peptide samples were dissolved in 2% acetonitrile/0.1%formic acid and analyzed using the Triple TOF 5600 plus mass spectrometer coupled with the Eksigent nano LC system (AB SCIEX, United States). The peptide solution was added to the C18 capture column (3 μM, 350 μM × 0.5 mm, AB Sciex, United States) and the C18 analytical column (3 μM, 75 μM × 150) was applied with a 60 min time gradient at a flow rate of 300 nl/min mm(Welch Materials, Inc.) for gradient elution. The two mobile phases were buffer A (2% acetonitrile/0.1%formic acid/98% H_2_O) and buffer B (98% acetonitrile/0.1% formic acid/2% H2O). For IDA (Information Dependent Acquisition), the MS spectrum was scanned with an anion accumulation time of 250 ms, and the MS spectrum of 30 precursor ions was acquired with an anion accumulation time of 50 ms. Collect MS1 spectrum in the range of 350–1200 m/z, and collect MS2 spectrum in the range of 100–1500 m/z. Set the precursor ion dynamic exclusion time to 15 s.

Genes identified from LC-MS and RNA-Seq were converted to ENTREZ ID and an R package, clusterProfiler, was used to perform gene ontology annotation (Yu et al., 2012). The mass spectrometry proteomics data have been deposited to the Proteome Xchange Consortium *via* the PRIDE (Perez-Riverol et al., 2022) partner repository with the dataset identifier PXD031350. RNA-Seq data were uploaded to the NCBI GEO database, numbered as GSE186861.

All the data needed to evaluate the conclusions are present in the paper and/or the supplementary information. All other relevant data are available from the authors upon reasonable request.

## Data Availability

The datasets presented in this study can be found in online repositories. The names of the repository/repositories and accession number(s) can be found below: Proteome Xchange Consortium *via* the PRIDE partner repository with the dataset identifier PXD031350; RNA sequencing data NCBI GEO accession: GSE186861.
